# Supplementary material for: MetaQTL: a package of new computational methods for the meta-analysis of QTL mapping experiments
Source: BMC Bioinformatics. 2007 Feb 8;8:49. doi: 10.1186/1471-2105-8-49 (PMC1808479; doi:10.1186/1471-2105-8-49)
Supplement: Additional File 7 — MetaQTL Package : jar file and tutorial. This Zip archive contains both the MetaQTL JAR file and the files of the tutorial. [file 1471-2105-8-49-S7.zip › org.inra.metaqtl/doc/org/metaqtl/bio/util/TabulatedWriter.html]

TabulatedWriter


|  |  |  |
| --- | --- | --- |
| **Overview** \| **Package** \| **Class** \| **Use** \| **Tree** \| **Deprecated** \| **Index** \| **Help** | |  |
| **PREV CLASS**   NEXT CLASS | **FRAMES**    **NO FRAMES**     **All Classes** |  |
| SUMMARY: NESTED \| FIELD \| CONSTR \| METHOD | DETAIL: FIELD \| CONSTR \| METHOD |  |


---


## org.metaqtl.bio.util Class TabulatedWriter

```
java.lang.Object
  org.metaqtl.bio.util.TabulatedWriter
```

---

``` public class TabulatedWriter extends java.lang.Object ```

General comments must be added here.

**Author:**
:   Jean-Baptiste
    Additions must be added here.

---

**Constructor Summary**

`TabulatedWriter(java.io.OutputStream stream)`

`TabulatedWriter(java.io.Writer writer)`


| **Method Summary** | |
| --- | --- |
| `void` | `close()` |
| `java.lang.String` | `getToken()` |
| `void` | `nextValues(java.util.Hashtable values)`             Prints one line of tabulated values given the hashtable which links values and keys. |
| `void` | `nextValues(java.lang.String[] values)`             Print one line of tabulated values by assuming that they are in the same order than the keys. |
| `void` | `setKeys(java.lang.String[] keys)` |
| `void` | `setToken(java.lang.String token)` |
| `int` | `size()` |

**Methods inherited from class java.lang.Object**

`clone, equals, finalize, getClass, hashCode, notify, notifyAll, toString, wait, wait, wait`

**Constructor Detail**

### TabulatedWriter

```
public TabulatedWriter(java.io.OutputStream stream)
```

---


### TabulatedWriter

```
public TabulatedWriter(java.io.Writer writer)
```


**Method Detail**

### getToken

```
public java.lang.String getToken()
```

:   **Returns:**: Returns the token.

---


### setToken

```
public void setToken(java.lang.String token)
```

:   **Parameters:**: `token` - The token to set.

---


### setKeys

```
public void setKeys(java.lang.String[] keys)
```

---


### nextValues

```
public void nextValues(java.util.Hashtable values)
```

:   Prints one line of tabulated values given the hashtable which links
    values and keys.

    :   **Parameters:**: `values` -

---


### nextValues

```
public void nextValues(java.lang.String[] values)
```

:   Print one line of tabulated values by assuming that they are in the same
    order than the keys.

    :   **Parameters:**: `values` -

---


### close

```
public void close()
           throws java.io.IOException
```

:   **Throws:**: `java.io.IOException`

---


### size

```
public int size()
```


---


|  |  |  |
| --- | --- | --- |
| **Overview** \| **Package** \| **Class** \| **Use** \| **Tree** \| **Deprecated** \| **Index** \| **Help** | |  |
| **PREV CLASS**   NEXT CLASS | **FRAMES**    **NO FRAMES**     **All Classes** |  |
| SUMMARY: NESTED \| FIELD \| CONSTR \| METHOD | DETAIL: FIELD \| CONSTR \| METHOD |  |


---
